# Cadaveric Analysis of Valvular Anatomy in the Great Saphenous Vein: Implications for Surgical Planning

**DOI:** 10.7759/cureus.87248

**Published:** 2025-07-03

**Authors:** Schafer Paladichuk, Alexander Downs, Zachary Brennan, Tianfu Shang, David Dommermuth, Steve Zeka, Dan Murphy, Ronald Walser, Tyler J Wallen

**Affiliations:** 1 Department of Anatomy, College of Osteopathic Medicine, Pacific Northwest University of Health Sciences, Yakima, USA; 2 Department of Cardiac Surgery, Smidt Heart Institute, Cedars-Sinai Medical Center, Los Angeles, USA; 3 Department of General Surgery, John A. Burns School of Medicine, University of Hawaii, Honolulu, USA; 4 Department of Emergency Medicine, Sutter Health, Roseville, USA; 5 Department of Cardiovascular Surgery, Geisinger Commonwealth School of Medicine, Wilkes-Barre, USA

**Keywords:** atherosclerosis, coronary artery bypass grafting, great saphenous vein, peripheral vascular bypass, thrombosis, valves

## Abstract

Introduction and aim

Coronary artery disease and peripheral vascular disease remain significant health concerns in the United States. If left untreated, surgical interventions like coronary artery bypass grafting (CABG) and peripheral vascular bypass (PVB) are frequently performed to restore vascular perfusion. The great saphenous vein (GSV) has historically been a primary conduit for these procedures. Complications involving the GSV include early-onset thrombosis and atherosclerosis, which are partly attributed to tunica intima valve flaps. These flaps can disrupt laminar flow even when the GSV is reversed. However, the GSV continues to play a crucial role in multi-vessel revascularization and PVB surgeries. This study aimed to analyze the GSV’s anatomical characteristics, particularly the distribution and spacing of its valves.

Methods

Cadaveric specimens were examined from 2021 to 2024. The GSV was incised, valve locations were marked and measured in relation to the inferior base of medial malleolus. Statistical analyses, including Student’s t-tests and ANOVA, were performed to assess differences.

Results

Results from 96 GSVs across 66 cadavers indicated an average of 5.7 valves per left GSV and 5.5 per right GSV. Significant differences were found between valve distributions above vs. below the knee (p<0.001), with increased inter-valve distance below the knee (p<0.001).

Conclusion

These data suggest that below knee segments may be more suitable for grafting because they have fewer valves and increased inter-valve distance. This study has the potential to provide insights for optimizing vein graft selection and improving surgical outcomes.

## Introduction

According to the 2016 Heart Disease and Stroke Statistics, coronary artery disease (CAD) and peripheral vascular disease (PVD) are highly prevalent diseases affecting more than 15.5 million and 10 million adults, respectively [1]. CAD and PVD are multifactorial with risk factors falling under either modifiable or nonmodifiable [2]. Modifiable risk factors include hypertension, smoking, obesity, and lipid profiles [2]. Nonmodifiable risk factors include gender, age, family history, and genetics [2]. Coronary artery bypass graft (CABG) and peripheral vascular bypass (PVB) are often part of the multidisciplinary treatment options for PVD and CAD [3,4]. CABG is the most commonly performed and studied cardiac procedure, with almost 400,000 procedures performed each year in the United States [3]. PVB has also become one of the most common vascular surgeries performed, with well over 100,000 each year [4]. The exact count of PVB procedures is unknown due to the lack of a single reporting center [4].

In 1967, Dr. René Favaloro first described the use of the autologous great saphenous vein (GSV) as a surgical bypass conduit due to its easy accessibility and significant length [1]. Commonly performed surgeries with the use of the GSV include CABG and PVBs [1,5-7]. Around 1986, data suggested the use of the left internal mammary artery (LIMA) was a superior vessel compared to the GSV, specifically for revascularization of the left anterior descending (LAD) artery during CABG [1]. Two of the most common and significant complications with GSV grafts are thrombosis and atherosclerosis [1,8]. However, certain cardiac surgery scenarios don’t allow for the preferred arterial grafts resulting in the GSV remaining the most commonly utilized second conduit [1,5,7]. In the context of PVBs, GSV remains the gold standard; however, other conduits such as alternative autologous veins, prosthetic, and cryopreserved vein grafts are available for use [9].

GSV vein mapping is an integral portion of arterial reconstruction due to the significant variance, vein quality, and distribution of valves. The more information known and presented on the GSV will lead to better outcomes for the patient [5-7]. There has been a lack of literature strictly assessing the GSV valve anatomy, warranting more studies [5-7]. This study aimed to characterize the anatomy of the GSV, with particular emphasis on valve distribution and inter-valvular spacing, to assess their potential impact on the quality and suitability of GSV segments used in surgical interventions.

## Materials and methods

**Figure 1:**
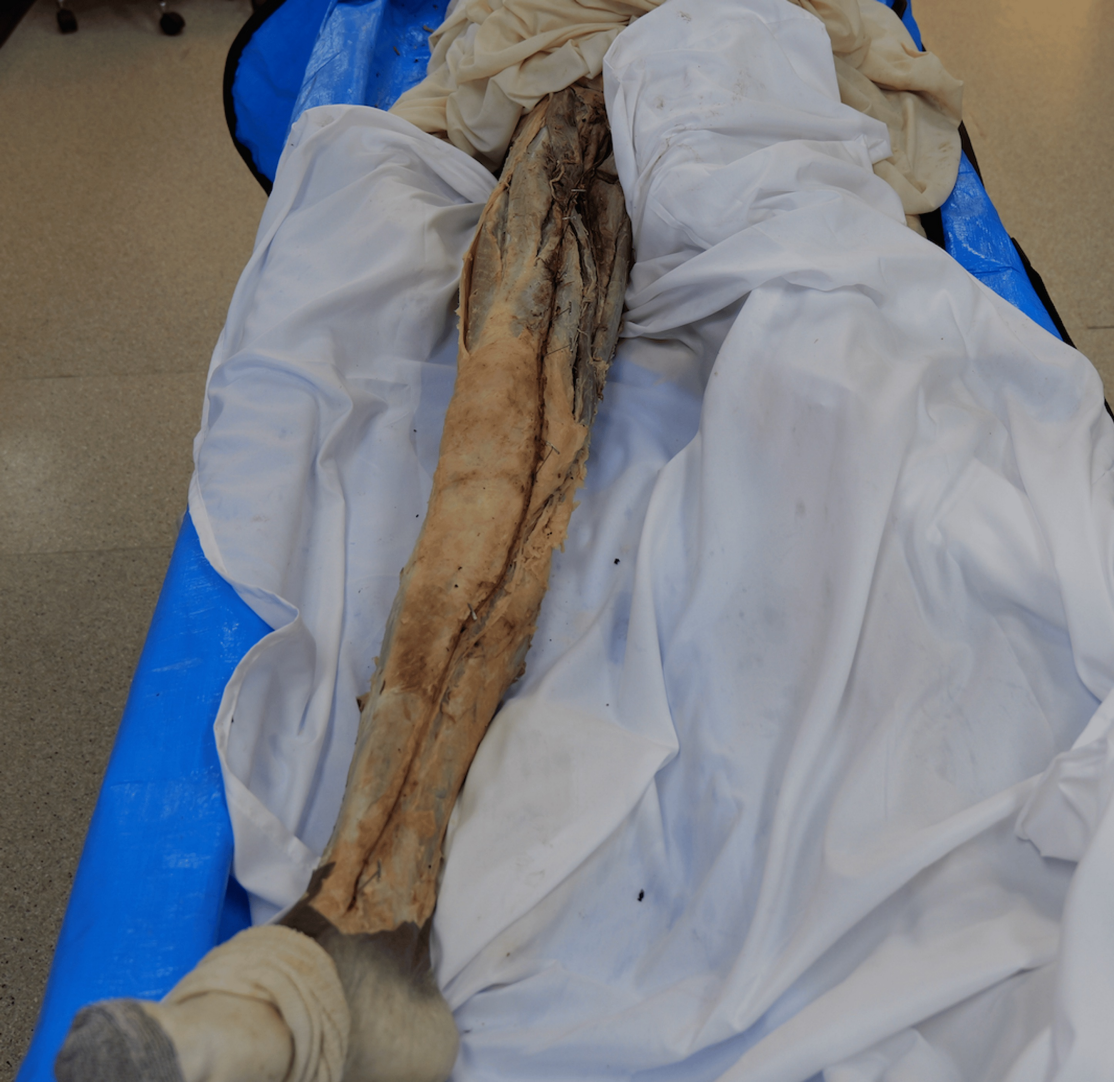
Gross image of great saphenous vein (GSV). Inferior view of the right leg demonstrating the GSV in full extension from the inferior border of the medial malleolus up to where it drains into the femoral vein at the saphenofemoral junction.

Cadavers from an academic institution (College of Osteopathic Medicine, Pacific Northwest University of Health Sciences) body donation program over the academic years from 2021 to 2024 were utilized. All donors had provided informed consent for the use of their bodies for scientific study and medical education prior to death. All rights of the donors were protected, and only the primary cause of death was available. Soft tissue dissection was conducted by the research team to isolate the GSV in its entirety. Inclusion criteria included a fully intact GSV, ability to incise the vessel, and a fully intact cadaveric limb (Figure 1). Exclusion criteria included disrupted vein integrity, limb amputation, and desiccation of the GSV that prevented proper incision and measurement.

**Figure 2:**
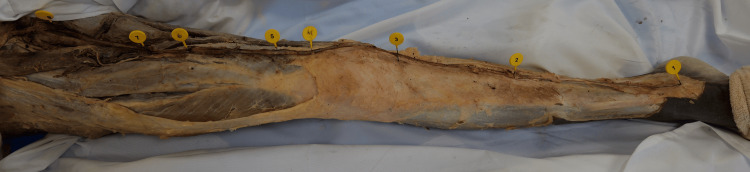
Gross image of great saphenous vein (GSV) with valve pins. Superior view of a right GSV with pins placed at all the valve locations.

The initial measurements of the GSV were conducted from the inferior base of the medial malleolus to the saphenofemoral junction (Figure 2). All measurements were taken with a standard measuring tape. Sharp scissors were used to make a single incision for the entire length of the GSV. Forceps were then used to glide superiorly, opening the vein. Pins were placed at every valve. Measurements were taken of the valves in relation to the inferior base of the medial malleolus (Figure 1). All measurements were confirmed by a minimum of two researchers and recorded in cm. Photographs were taken of the veins and valves. Statistical analysis performed included Student’s t-test, along with one-way ANOVA and Tukey’s HSD, which were used to determine significance (p<0.05).

Analysis of the data was performed using SAS version 9.4 (Cary, NC: SAS Institute Inc.). For the Student’s t-test, the GSV was divided into above-knee and below-knee sections. This division was chosen for simplicity, as endoscopic harvest of the GSV typically involves placing the endoscopic trocar just below the medial tibial plateau [10]. Thus, segments proximal to the port site were classified as above knee, and those distal to it as below knee. Further, a series of one-way ANOVAs with Tukey’s Honestly Significant Difference (HSD) post-test adjustments were performed to compare the mean number and distance between valves in the proximal, middle, and distal regions of the GSV. Dividing the GSV into three sections was a way for the researcher to further refine the section of the GSV with the least number of valves and increased inter-valve distance.

## Results

Characteristics of the specimen sample are shown in Table 1. The sample comprised 96 veins, 50 from the right leg and 46 from the left, from 66 adult cadavers. Of these cadavers, 39 (59%) were female and 27 (41%) were male. As shown in Table 1, the mean number of valves in the great saphenous vein (GSV), measured from the medial malleolus to the saphenofemoral junction, was 5.7 overall - 5.6 in the left leg and 5.4 in the right for females, and 5.5 in the left leg and 5.4 in the right for males. The number of valves ranged from a minimum of 2 to a maximum of 13.

**Table 1:** Specimen sample characteristics. Data are presented as mean (SD). GSV: great saphenous vein; N: vein quantity

Characteristic	Female, N=39	Male, N=27
Left GSV		
Length (cm)	76.6 (4.7)	80.5 (5.8)
Number of valves	5.7 (2.3)	5.6 (2.2)
Distance between valves (cm)	9.5 (7.6)	10.5 (8.1)
Right GSV		
Length (cm)	75.8 (4.3)	81.2 (6.4)
Number of valves	5.5 (2.5)	5.4 (1.9)
Distance between valves (cm)	10.4 (8.9)	11.1 (7.8)

Above- and below-knee regions

**Figure 3:**
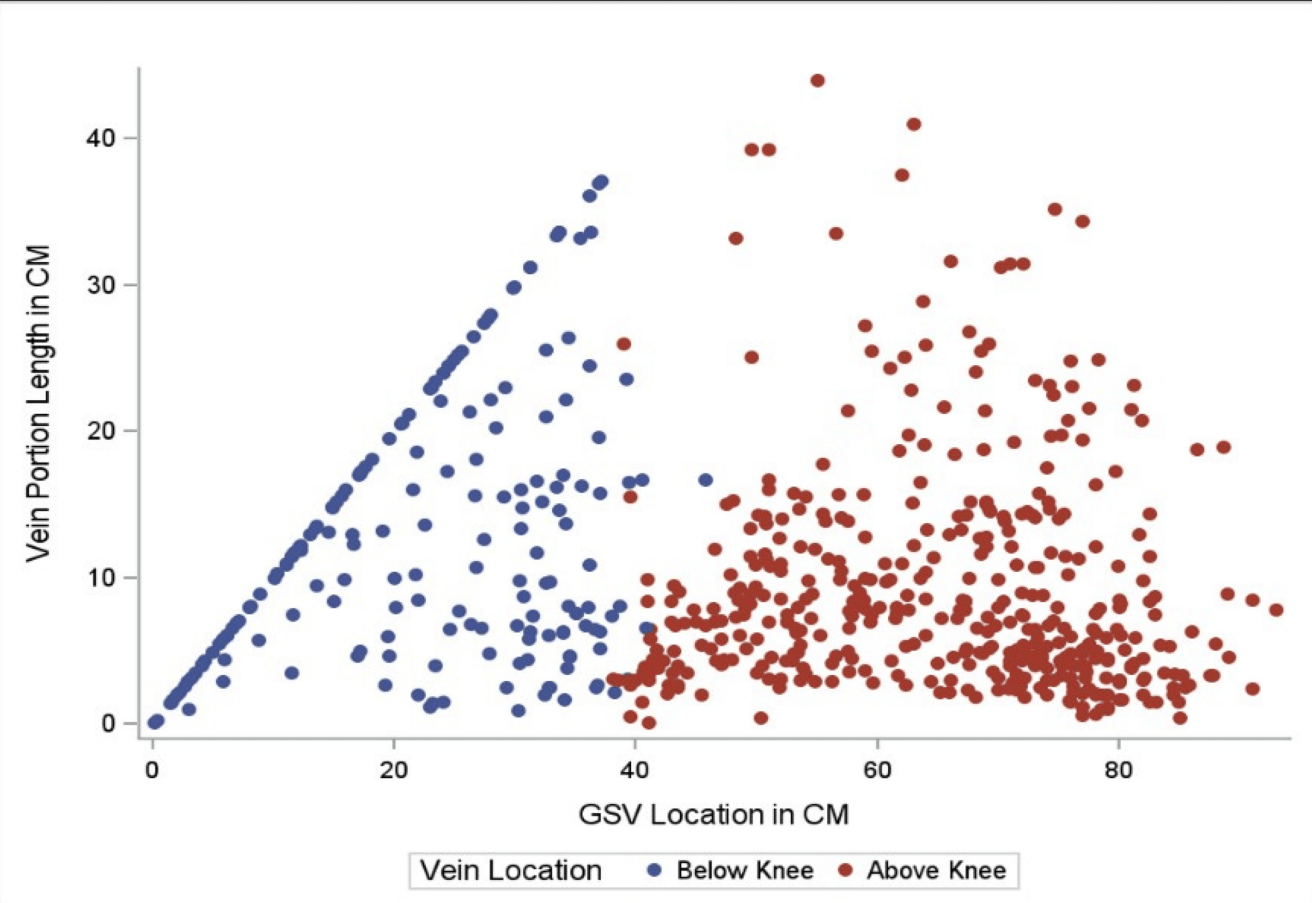
Valve-free length by GSV location. Scatter plot looking at inter-valve distance and location of the GSV valves. Data points are divided into the following two sections: below knee and above the knee. Clustered dot points are consistent with more valves in that section of GSV and decreased inter-valve distance. More space between data points indicates fewer valves in the area with greater inter-valve distance. GSV: great saphenous vein

**Figure 4:**
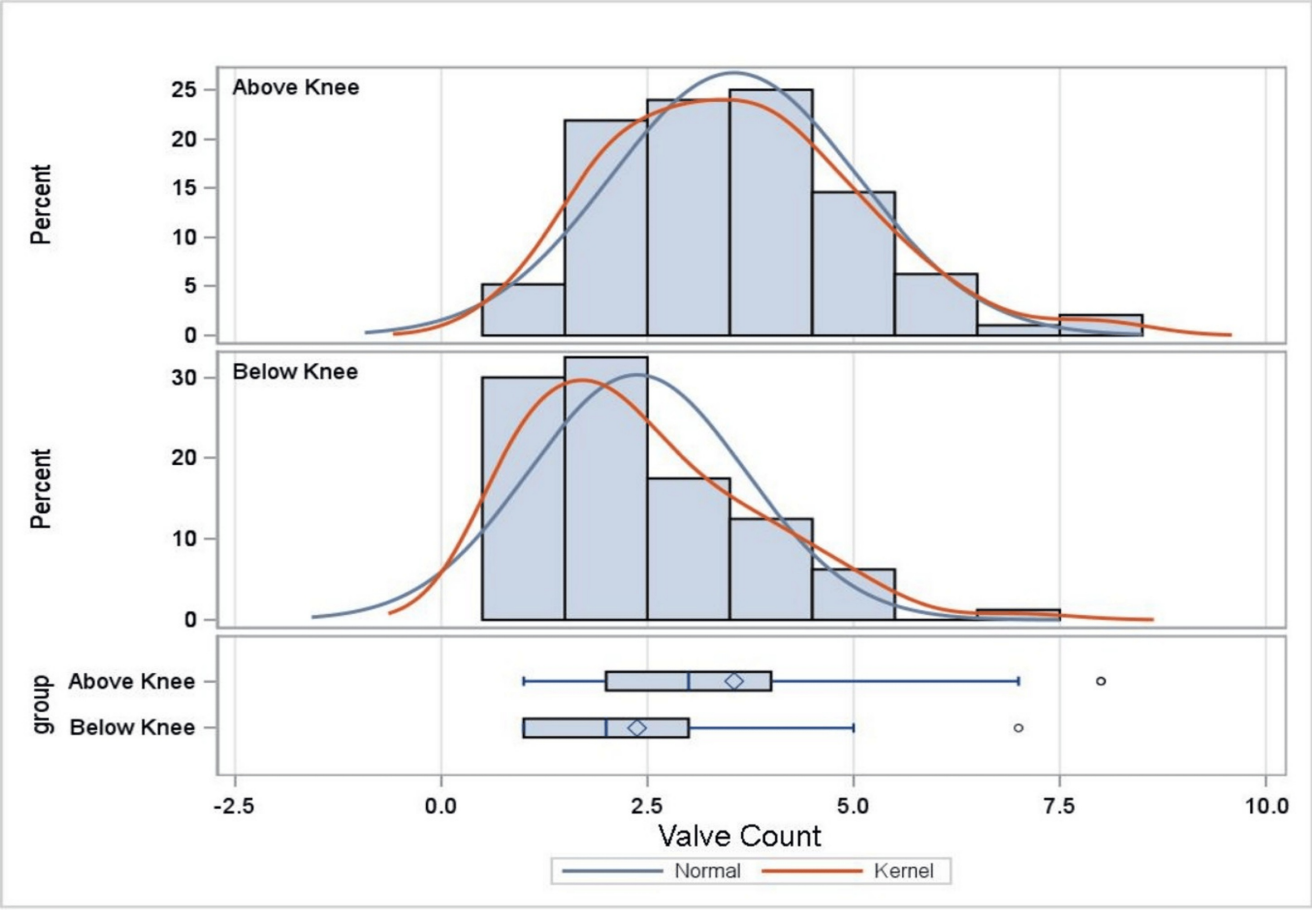
Distribution of GSV valves. Bar graph and box and whisker plot demonstrating valve quantification in the above knee and below knee sections. Y-axis demonstrates the percentage of valve quantities above and below the knee sections. X-axis demonstrates GSV quantities. Kernel represents the estimated distribution of the cadaveric GSV vein valve distribution. GSV: great saphenous vein

**Figure 5:**
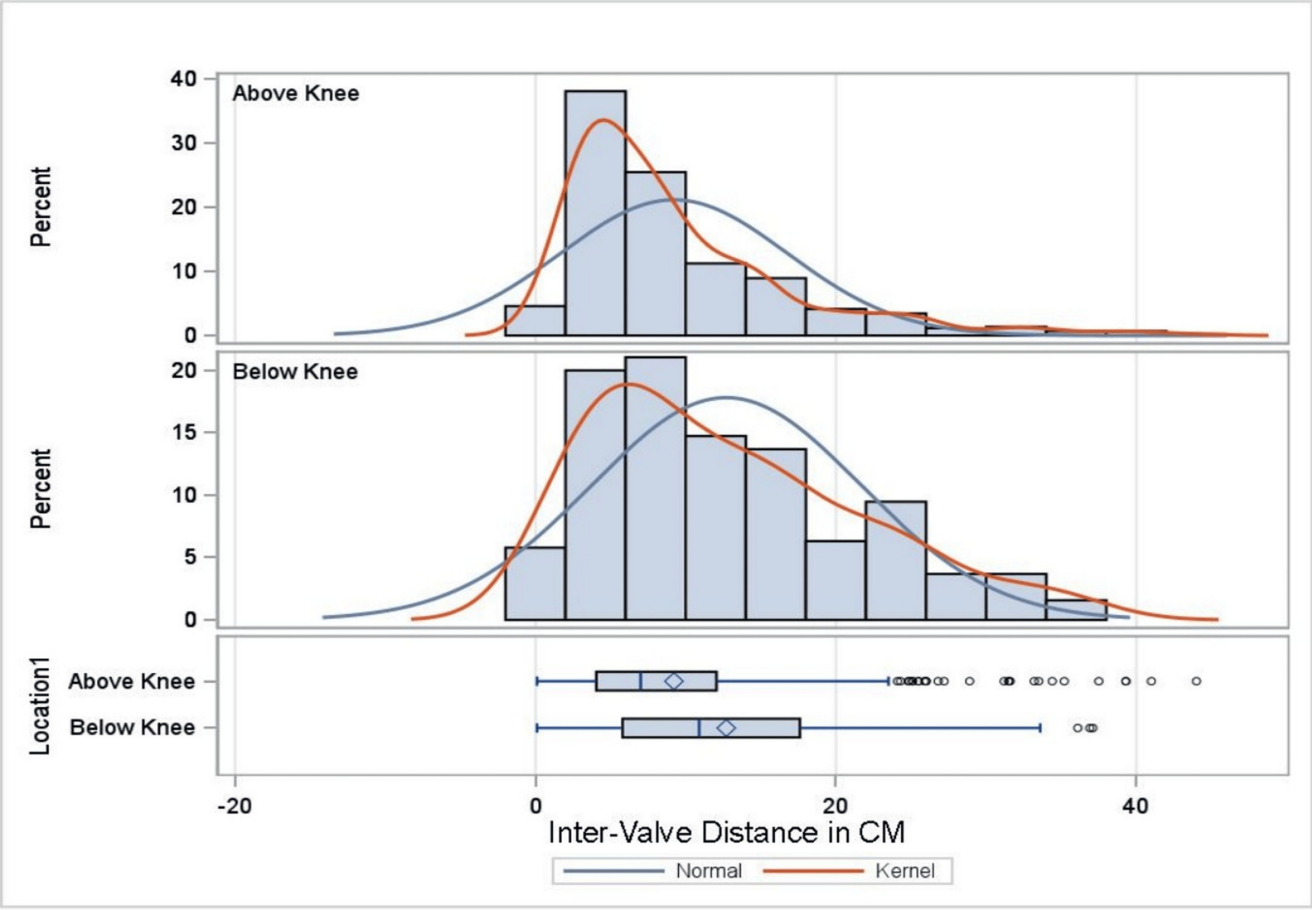
Distribution of GSV inter-valve distance. Bar graph and box and whisker plot demonstrating inter-valve distance between the following two sections: below knee and above knee. Y-axis demonstrates the percentage of valves documented at that inter-valve length. X-axis demonstrates inter-valve length in cm. Both above the knee and below the knee demonstrate a right tail. Kernel represents the estimated distribution of the cadaveric inter-valve distance within the GSV. GSV: great saphenous vein

As shown in Table 2, the difference between the mean number of valves above and below the knee is statistically significant, with the below-knee section containing fewer valves (t {174}=5.49, p<0.001). Further, the mean distance between valves above and below the knee is also statistically significant, with the below-knee section having increased inter-valve distance (t {624} = -5.01, p<0.001). This information is further supported by Figures 3-5, which demonstrate, with statistical significance, that increased inter-valve distance and decreased valve quantity are observed in the below-knee sections.

**Table 2:** Student’s t-test demonstrating statistically significant differences in mean number and distance between valves located below the knee region of the GSV. Mean differences are significant at the p<0.05 level. GSV: great saphenous vein

Student’s t-test					
Measure	Region I	Region J	Mean difference I-J (95% CI)	t-Statistic	p-Value
Number of valves	Above knee	Below knee	1.18 (0.75 to 1.60)	5.49	<0.001
Distance between valves (cm)	Above knee	Below knee	-3.49 (-4.85 to -2.12)	-5.01	<0.001

Proximal, middle, and distal regions 

Analysis results reveal statistically significant differences over the proximal, middle, and distal regions of the GSV in mean number (F=17.45, p<0.001) and distance between valves (F=8.44, p<0.001). As shown in Table 3, Tukey’s HSD test for multiple comparisons finds the mean number of valves to be significantly different between the proximal and distal regions (p<0.001, 95% CI=0.67, 1.34), proximal and middle regions (p=0.003, 95% CI=0.16, 0.78), and middle and distal regions of the GSV (p=0.002, 95% CI=0.19, 0.88). In addition, Tukey’s HSD test for multiple comparisons finds the mean distance between valves to be significantly different between the proximal and distal regions (p<0.001, 95% CI = -4.38, -1.38) and proximal and middle regions of the GSV (p=0.001, 95% CI = -2.84, -0.41). Overall, the data demonstrate, with statistical significance, that the distal region has the fewest mean number of valves and the greatest inter-valve distance (in cm) (Figures 3-5).

**Table 3:** One-way ANOVA with Tukey's HSD demonstrating statistically significant differences among the mean number and distance between valves in the distal region of the GSV. Mean differences are significant at the p<0.05 level. GSV: great saphenous vein; HSD: Honestly Significant Difference

One-way ANOVA with Tukey’s HSD					
Measure	Region I	Region J	Mean difference I-J (95% CI)	f-Value	p-Value
Number of valves	Proximal	Distal	1.01 (0.67 to 1.34)	17.45	<0.001
	Proximal	Medial	0.47 (0.16 to 0.78)	1.41	0.003
	Medial	Distal	0.54 (0.19 to 0.88)	8.30	0.002
Distance between valves (cm)	Proximal	Distal	-2.88 (-4.38 to -1.38)	8.44	<0.001
	Proximal	Medial	-1.63 (-2.84 to -0.41)	7.31	0.001

## Discussion

Despite well-known complications, such as thrombosis and atherosclerosis, which are among the most common and significant issues associated with a GSV graft, the great saphenous vein remains a commonly used conduit, even when compared to its arterial counterparts [1]. These complications have been estimated to occur in as many as 10-25% of cases within 12-18 months postoperatively [1,8,10]. There is an additional 5% increase in conduit failure rate for each year past the five-year post-bypass mark [1,8,10]. Increased graft failure has been linked to the venous conduit being introduced into an arterial environment, valves promoting turbulent flow (even in the reverse graft setting), and intimal hyperplasia secondary to endothelial injury [1,8,10]. The data presented here suggest fewer valves with increased inter-valve distance are most consistently seen in the below-knee section, more specifically, in the distal section of the GSV. Thus, this has the potential to be a favorable section for harvest due to decreased potential of turbulent blood flow. If this section of the GSV is consistently used during surgical intervention, this has the possibility of resulting in fewer complications, including both atherosclerosis and thrombosis.

This study represents the largest known cadaveric analysis of valve distribution in the great saphenous vein (GSV) to date and is consistent with the findings of Portugal et al., who examined valve distribution in 30 cadaveric specimens [7]. Other investigations, such as those by Tepelenis et al. and Nakahara et al., have explored related anatomical features, including GSV tributaries, variations, and the use of imaging modalities like computed tomography to identify valves preoperatively for improved graft outcomes [11,12].

It is important to note that this study focused exclusively on the quantity of valves and inter-valve distances within the GSV. However, another critical characteristic for conduit suitability is vein diameter. A diameter greater than 3.0 mm has been associated with improved long-term patency and durability, which may limit the utility of the below-knee segment [13]. Future iterations of this study aim to incorporate measurements of vein diameter at each marked valve, enabling more precise identification of optimal GSV segments for grafting. Clinically, the preferential use of the below-knee segment may also increase the risk of wound healing complications or infections, particularly in patients with peripheral vascular disease (PVD). These considerations further underscore the need for a personalized approach to vein conduit selection.

Several limitations should be considered when interpreting the findings of this study. First, the use of cadaveric specimens may limit results as the embalming process can alter valve structure. Some cadaveric specimens had only one viable GSV available for analysis, resulting in an uneven distribution of cadavers to GSVs used; this may limit the generalizability of the findings. Additionally, the demographic characteristics of the cadaver population, primarily elderly individuals with an average age exceeding 75 years, may not be representative of the broader patient population undergoing bypass procedures. The cause of death and medical history, including the presence of vascular disease, were not available, which may have introduced unrecognized variability in vein quality. Furthermore, the study did not assess GSV diameter, wall thickness, or branching patterns, all of which are relevant to graft suitability. Finally, this study was conducted at a single academic institution, limiting generalizability.

## Conclusions

Integrating the findings of this study with the broader body of literature may enhance our understanding of GSV morphology and its surgical implications. Notably, the data from this and similar studies are most impactful during preoperative planning, where patient-specific conduit selection may enhance graft success and longevity. Future research should incorporate assessments of GSV diameter at valve sites, branching patterns, and harvest techniques to provide a more comprehensive understanding of the venous conduit and its role in optimizing surgical outcomes.
